# Thrombin Promotes Matrix Metalloproteinase-13 Expression through the PKC**δ**/c-Src/EGFR/PI3K/Akt/AP-1 Signaling Pathway in Human Chondrocytes

**DOI:** 10.1155/2013/326041

**Published:** 2013-12-09

**Authors:** Chun-Yin Huang, Hsiu-Jung Lin, Hsin-Shui Chen, Shi-Yann Cheng, Horng-Chaung Hsu, Chih-Hsin Tang

**Affiliations:** ^1^Graduate Institute of Clinical Medical Science, China Medical University, Taichung, Taiwan; ^2^Department of Orthopaedic Surgery, China Medical University Beigang Hospital, Yunlin County, Taiwan; ^3^School of Medicine, China Medical University, Taichung, Taiwan; ^4^Department of Nursing, China Medical University Beigang Hospital, Yunlin County, Taiwan; ^5^Department of Physical Medicine and Rehabilitation, China Medical University Beigang Hospital, Yunlin County, Taiwan; ^6^Department of Obstetrics and Gynecology, China Medical University Beigang Hospital, Yunlin County, Taiwan; ^7^Department of Orthopaedic Surgery, China Medical University Hospital, Taichung, Taiwan; ^8^Graduate Institute of Basic Medical Science, China Medical University, Taichung, Taiwan; ^9^Department of Biotechnology, College of Health Science, Asia University, Taichung, Taiwan

## Abstract

Thrombin is a key mediator of fibrin deposition, angiogenesis, and proinflammatory processes. Abnormalities in these processes are primary features of rheumatoid arthritis and osteoarthritis. Matrix metalloproteinase-13 (MMP-13) may contribute to the breakdown of articular cartilage during arthritis. However, the role of thrombin in MMP-13 production in chondrocytes is unknown. In this study, we investigated the intracellular signaling pathways involved in thrombin-induced MMP-13 expression in human chondrocytes. We found that stimulation with thrombin led to increased secretion of MMP-13 in cultured human chondrocytes. Further, this thrombin-induced MMP-13 production was reduced after transfection with siRNAs against protease activated receptors 1 and 3 (PAR1 and PAR3), but not with PAR4 siRNA. Treatment with specific inhibitors for PKC**δ**, c-Src, EGFR, PI3K, Akt, or AP-1 or with the corresponding siRNAs against these signaling proteins also abolished the thrombin-mediated increase in MMP-13 production in chondrocytes. Our results provide evidence that thrombin acts through the PAR1/PAR3 receptors and activates PKC**δ** and c-Src, resulting in EGFR transactivation and activation of PI3K, Akt, and finally AP-1 on the MMP-13 promoter, thereby contributing to cartilage destruction during arthritis.

## 1. Introduction

Chondrocytes are the only cellular components in cartilage and they maintain an equilibrium between anabolic and catabolic activities, which are necessary for preservation of the structural and functional integrity of the tissue during normal physiological conditions [1]. Under normal conditions, chondrocytes express various proteolytic enzymes such as aggrecanases and matrix metalloproteinases (MMPs), which mediate the very low matrix turnover that is responsible for cartilage remodeling [2]. In contrast, in pathological conditions such as osteoarthritis (OA) or rheumatoid arthritis (RA), chondrocytes increase the production of these enzymes considerably, resulting in aberrant cartilage destruction [3, 4]. Therefore, understanding the molecular mechanisms regulating the expression of these enzymes and identification and specific targeting of critical signaling effectors will help develop better treatment strategies for OA and RA.

MMPs are a large family of structurally related calcium- and zinc-dependent proteolytic enzymes involved in the degradation of different components of the extracellular matrix [5]. MMPs are expressed in a number of different cell types and play a key role in diverse cellular processes [6]. Among the MMPs, MMP-13 (collagenase-3) actively degrades type II collagen, the major collagen type in the cartilage, and hence is of particular interest because of its role in cartilage degradation [7, 8]. It has been previously shown that MMP-13 is overexpressed in OA and RA [9] and recent reports provide evidence that anti-MMP-13 therapy is a promising new strategy for treatment of arthritis [8]. Given their important role in cellular functions, MMPs are tightly regulated at multiple levels, that is, through regulation of gene transcription, protein synthesis, and the extracellular activities of MMPs. Complete understanding of the various factors and pathways involved in the regulation of MMP expression is important in the context of developing potential therapies.

Thrombin is a multifunctional protease that can activate hemostasis and coagulation through the cleavage of fibrinogen to form fibrin clots [10]. Increase in fibrin deposition, which contributes to chronic inflammation and progressive tissue abnormalities, is a predominant feature of OA and RA [11]. Thrombin also acts as a mitogen to stimulate abnormal proliferation of synovial cells during OA and RA pathogenesis [12, 13]. Thrombin activates intracellular signaling pathways by interacting with the transmembrane domains of G-protein-coupled receptors (GPCR), known as protease activated receptors (PARs). Four members have been cloned and have been designated PAR1, PAR2, PAR3, and PAR4 [14]. Three of these members, PAR1, PAR3, and PAR4, are cleaved by thrombin, whereas PAR2 is cleaved by trypsin. The various physiological or pathogenic effects of thrombin are due to the widespread expression of thrombin receptors in many cells [15]. Increase in thrombin receptor mRNA in arthritis has been reported [16].

Synovium may be involved in the induction of catabolic activities in the cartilage of the joints in OA and RA pathogenesis. Upon stimulation, chondrocytes in the cartilage of the joints release matrix-degradation enzymes such as MMP-13, which results in the destruction of cartilage [3]. Thrombin is known to play an important role in both OA and RA [17, 18]. However, the effect of thrombin on MMP-13 expression in human chondrocytes is unknown. In this study, we found that thrombin increased the expression of MMP-13 in cultured chondrocytes. In addition, the PAR1/PAR3 receptor, PKC*δ*, c-Src, EGFR transactivation, and the PI3K/Akt and AP-1 signaling pathways may be involved in the thrombin-induced increase of MMP-13 expression. These results provide new insights into the mechanisms of thrombin action, which could be of therapeutic value in the treatment of arthritis.

## 2. Materials and Methods

### 2.1. Materials

Anti-mouse IgG and anti-rabbit IgG-conjugated horseradish peroxidase, rabbit polyclonal antibodies specific for *β*-actin, MMP-13, phospho (p)-c-Src, c-Src, PKC*δ*, EGFR, p-p110, p110, p-Akt, Akt, p-c-Jun, and c-Jun were purchased from Santa Cruz Biotechnology (Santa Cruz, CA). Rabbit polyclonal antibodies specific for p-PKC*δ* and p-EGFR were purchased from Cell Signaling and Neuroscience (Danvers, MA). The MMP-13 enzyme immunoassay kit was purchased from R&D Systems (Minneapolis, MN, USA). SFLLRN-NH_2_ (a PAR1 agonist peptide), TFRGAP-NH_2_ (a PAR3 agonist peptide), and GYPGQV-NH_2_ (a PAR4 agonist peptide) were purchased from Bachem. The AP-1 luciferase plasmid was purchased from Stratagene (La Jolla, CA). The pSV-*β*-galactosidase vector and luciferase assay kit were purchased from Promega (Madison, WI). All other chemicals were obtained from Sigma-Aldrich (Saint Louis, MO).

### 2.2. Cell Cultures

Primary cultures of human chondrocytes were isolated from articular cartilage as previously described [19, 20]. After approval by the institutional ethics committee, human articular chondrocytes were isolated during the knee replacement surgeries of patients with OA. Cartilage pieces were minced finely, and chondrocytes were isolated by sequential enzymatic digestion at 37°C with 0.1% hyaluronidase for 30 min and then with 0.2% collagenase for 1 h. Isolated chondrocytes were filtered through 70-*μ*M nylon filters. The cells were grown on plastic cell culture dishes in Dulbecco's modified Eagle medium (DMEM; Gibco, Grand Island, NY) supplemented with 20 mM HEPES and 10% heat-inactivated FBS, 2 mM glutamine, 100 U/mL penicillin, and 100 *μ*g/mL streptomycin (pH adjusted to 7.6) at 5% CO_2_.

### 2.3. Measurement of MMP-13 Production

Human chondrocytes (2 × 10^4^) were cultured in 24-well culture plates. Cells were incubated with thrombin for 24 h at 37°C. After incubation, the medium was removed and stored at –80°C until the assay. MMP-13 in the medium was assayed using the MMP-13 enzyme immunoassay kits, as per the manufacturer's instructions.

### 2.4. Quantitative Real-Time PCR

Total RNA was extracted from chondrocytes with a TRRzol kit (MDBio Inc., Taipei, Taiwan). The reverse transcription reaction was performed using 2 *μ*g of total RNA (in 2 *μ*L of RNase-free water) that was reverse transcribed into cDNA with an MMLV RT kit (Promega, Madison, WI) by following the manufacturer's instructions [21]. The reverse transcription reaction mixture was incubated at 37°C for 60 min and then at 70°C for 5 min to inactivate MMLV. Quantitative real-time PCR (qPCR) analysis was carried out with TaqMan One-Step PCR Master Mix (Applied Biosystems, Foster City, CA). cDNA template (2 *μ*L) was added to each 25 *μ*L reaction with sequence-specific primers and TaqMan probes. All target gene primers and probes were purchased commercially (*β*-actin was used as an internal control) (Applied Biosystems). qPCR assays were carried out in triplicate on a StepOnePlus sequence detection system. The cycling conditions were as follows: 10 min polymerase activation at 95°C followed by 40 cycles at 95°C for 15 s and at 60°C for 60 s. The threshold was set above the nontemplate control background and within the linear phase of target gene amplification to calculate the cycle number at which the transcript was detected (denoted *C*_*T*_).

### 2.5. Western Blot Analysis

Cellular lysates were prepared as previously described [22]. Proteins were resolved using SDS-PAGE and transferred to Immobilon polyvinyldifluoride membranes. The membranes were blocked with 4% BSA for 1 h at room temperature and then probed with rabbit antibodies against human p-110, p110, p-Akt, Akt, p-c-Jun, or c-Jun (1 : 1000) for 1 h at room temperature. After 3 washes, the blots were incubated with a donkey anti-rabbit peroxidase-conjugated secondary antibody (1 : 1000) for 1 h at room temperature. The blots were visualized with enhanced chemiluminescence on Kodak X-OMAT LS film (Eastman Kodak, Rochester, NY).

### 2.6. Kinase Activity Assay

PKC*δ* and c-Src activity were assessed with the PKC*δ* kinase activity assay kit (Assay Designs, MI) and the c-Src kinase activity assay kit (Abnova, Taipei, Taiwan), respectively. The kinase activity kits are based on a solid-phase ELISA that uses a specific synthetic peptide as a substrate for PKC*δ* or c-Src and a polyclonal antibody that recognizes the phosphorylated form of the substrate.

### 2.7. Transfection of siRNAs

ON-TARGETplus siRNA targeting PAR1, PAR3, PAR4, PKC*δ*, c-Src, EGFR, p110, Akt, c-Jun, and control were purchased from Dharmacon Research (Lafayette, CO, USA). Transient transfection of siRNAs was carried out using DharmaFECT1 transfection reagent. siRNA (100 nM) was formulated with DharmaFECT1 transfection reagent according to the manufacturer's instructions.

### 2.8. Transfection and Reporter Gene Assay

Cells were cotransfected with 0.8 *μ*g AP-1 luciferase plasmid and 0.4 *μ*g *β*-galactosidase expression vector. Cells were grown to 80% confluency in 12-well plates and then transfected on the following day with Lipofectamine 2000 (LF2000; Invitrogen). DNA and LF2000 were premixed for 20 min and then added to the cells. After 24 h, the cells were incubated with the indicated reagents. After a further 24 h of incubation, the medium was removed, and cells were washed once with cold PBS. To prepare lysates, 100 *μ*L reporter lysis buffer (Promega, Madison, WI) was added to each well, and cells were scraped from the dishes. The supernatant was collected after centrifugation at 13,000 rpm for 2 min. Aliquots of cell lysates (20 *μ*L) containing equal amounts of protein (20–30 *μ*g) were placed into wells of an opaque black 96-well microplate. An equal volume of luciferase substrate was added to all samples, and luminescence was measured in a microplate luminometer. The value of luciferase activity was normalized to the transfection efficiency, which was monitored by activity of the cotransfected *β*-galactosidase expression vector.

### 2.9. Chromatin Immunoprecipitation Assays

Chromatin immunoprecipitation analyses were performed as described previously [23]. DNA was immunoprecipitated with an anti-c-Jun antibody and purified and extracted with phenol-chloroform. The purified DNA was quantified by quantitative real-time PCR and normalized with the input DNA, which was performed in triplicate with SYBR green mix using the StepOnePlus sequence detection system. The primers 5′-AACAAGAGATGCTCTCA-3′ and 5′-TGAATGGTGATGCCTGG-3′ were used to amplify the human MMP-13 promoter region (from –182 to +27) [9].

### 2.10. Statistics

Data were expressed as means ± SEM. For statistical evaluation, we used the Mann-Whitney *U* test for non-Gaussian parameters. The difference was considered significant if the *P* value was <0.05.

## 3. Results

### 3.1. Thrombin Induces MMP-13 Expression in Human Chondrocytes

**Figure 1:**
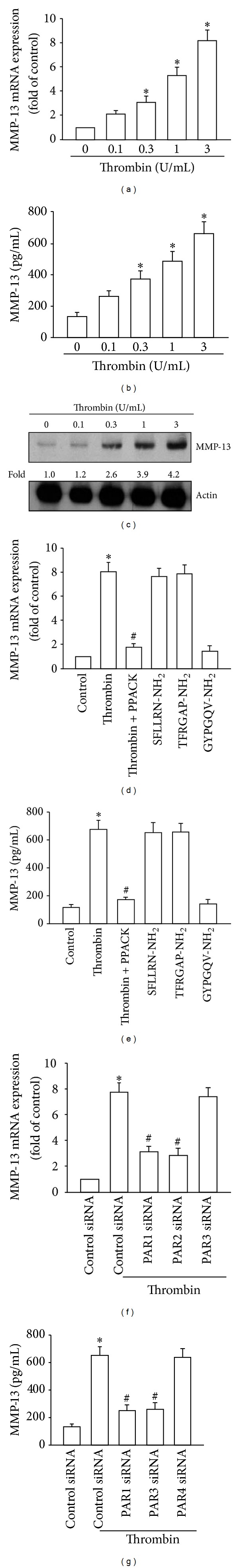
Involvement of PAR1/PAR3 receptor in thrombin-induced MMP-13 expression in human chondrocytes (a) qPCR analyses of MMP-13 mRNA and (b) and (c) ELISA and Western blotting analyses of protein expression of MMP-13 in chondrocytes incubated with thrombin for 24 h. (d) and (e) qPCR and ELISA analyses of MMP-13 expression in chondrocytes treated with thrombin alone (3 U/mL), thrombin + PPACK (30 nM), SFLLRN-NH_2_ (100 *μ*M), thrombin + TFRGAP-NH_2_ (100 *μ*M), or thrombin + GYPGQV-NH_2_ (100 *μ*M) for 24 h. (f) and (g) qPCR and ELISA analyses of MMP-13 expression in chondrocytes, which were transfected with siRNAs against PAR1, PAR3, PAR4, or control siRNA for 24 h and then stimulated with thrombin for 24 h. *P < 0.05 as compared with basal level. ^#^ *P* < 0.05 as compared to the levels in the thrombin-treated group.

The levels of clotting factors and fibrinolytic products such as thrombin increase in patients with arthritis [24]. In addition, MMP-13 has been reported to participate actively in the destruction of cartilage [9]. Therefore, we investigated the effect of thrombin on MMP-13 expression in human chondrocytes. Stimulation of cells with thrombin (0.1–3 U/mL) increased the mRNA expression of MMP-13 dose dependently (Figure 1(a)). Thrombin also increased the protein expression of MMP-13 in chondrocytes in a concentration-dependent manner as assessed using ELISA assays and Western blotting analyses (Figures 1(b) and 1(c)). To confirm that the effect was thrombin induced, we used PPACK, a thrombin inhibitor. Pretreatment of cells with PPACK led to significant antagonization of the potentiating effect of thrombin on MMP-13 expression (Figures 1(d) and 1(e)). These data indicate that thrombin increases MMP-13 expression in human chondrocytes.

### 3.2. Involvement of PAR1/PAR3 Receptors in Thrombin-Induced MMP-13 Expression in Human Chondrocytes

Thrombin has been reported to exert its effects through specific interactions with PAR1, PAR3, and PAR4 receptors [25, 26]. To determine the role of PAR-dependent signaling in the regulation of MMP-13 production in chondrocytes, cells were treated with PAR1-, PAR3-, or PAR4-specific agonist peptides and then the expression levels of MMP-13 were examined. Stimulation of cells with SFLLRN-NH_2_ (PAR1 agonist peptide; 100 *μ*M) or TFRGAP-NH_2_ (PAR3 agonist peptide; 100 *μ*M), but not GYPGQV-NH_2_ (PAR4 agonist peptide; 100 *μ*M), increased MMP-13 mRNA and protein expression (Figures 1(d) and 1(e)). To verify that PAR1 and PAR3 subtype receptors were involved in the thrombin-mediated increase of MMP-13 expression, PAR receptor expression was specifically inhibited with siRNA. Transfection of cells with PAR1 or PAR3 siRNA, but not PAR4 siRNA, abolished the thrombin-stimulated increase in MMP-13 production (Figures 1(f) and 1(g)). These data suggest that the thrombin-PAR1/PAR3 interactions were important for MMP-13 production in human chondrocytes.

### 3.3. The PKC*δ* and c-Src Signaling Pathways Mediate Thrombin-Induced MMP-13 Expression

**Figure 2:**
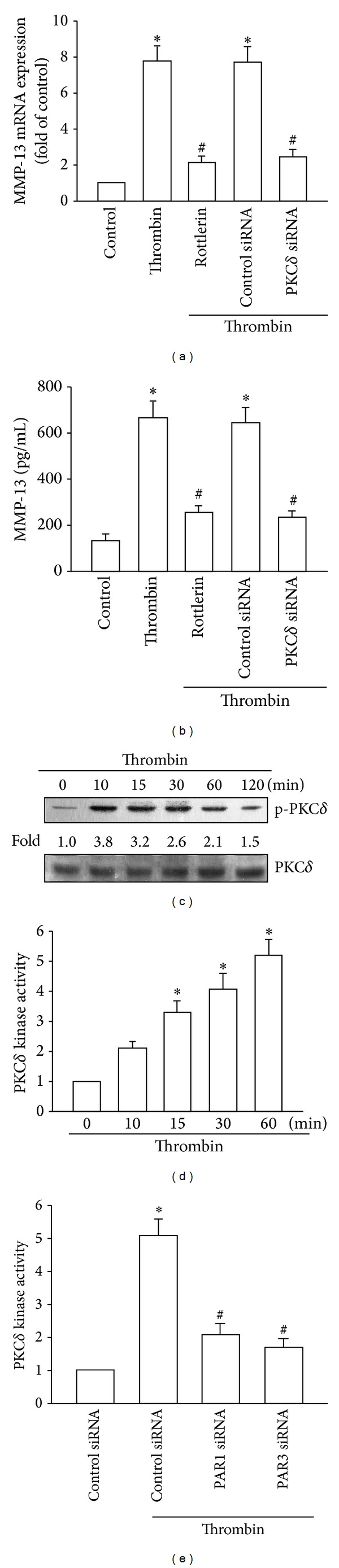
Role of PKC*δ* in thrombin-induced MMP-13 production. ((a) and (b)) qPCR and ELISA analyses of MMP-13 expression in chondrocytes, which were pretreated with rottlerin (3 *μ*M) for 30 min or transfected with siRNA against PKC*δ* for 24 h and then stimulated with thrombin for 24 h. ((c) and (d)) Western blotting analysis of phosphorylation and kinase activity of PKC*δ* in cells incubated with thrombin for the indicated time intervals. (e) Kinase activity of PKC*δ* in chondrocytes, which were transfected with siRNA against PAR1 or PAR3 for 24 h and stimulated with thrombin for 60 min. *P < 0.05 as compared with basal levels. ^#^ *P* < 0.05 as compared with the levels in the thrombin-treated group.

PKC*δ*-dependent c-Src activation has been shown to play an important role in thrombin-induced gene expression in human osteoblasts and synovial fibroblasts [25, 27]. To determine whether PKC*δ* is involved in thrombin-mediated MMP-13 expression, the selective PKC*δ* inhibitor rottlerin [28] was used. Pretreatment of cells with rottlerin diminished thrombin-induced MMP-13 expression (Figures 2(a) and 2(b)), suggesting that PKC*δ* plays a potential role in thrombin-induced MMP-13 production. Transfection of cells with PKC*δ*-specific siRNA also led to reduction in thrombin-induced MMP-13 expression (Figures 2(a) and 2(b)). Next, PKC*δ* phosphorylation and activity in cells stimulated with thrombin were determined. Stimulation of cells with thrombin promoted PKC*δ* phosphorylation and kinase activity (Figures 2(c) and 2(d)). In addition, thrombin-mediated PKC*δ* kinase activity was abolished in cells transfected with PAR1 or PAR3 siRNA (Figure 2(e)). Therefore, thrombin induces MMP-13 expression through the PAR1/PAR3–PKC*δ* signaling pathway in human chondrocytes.

**Figure 3:**
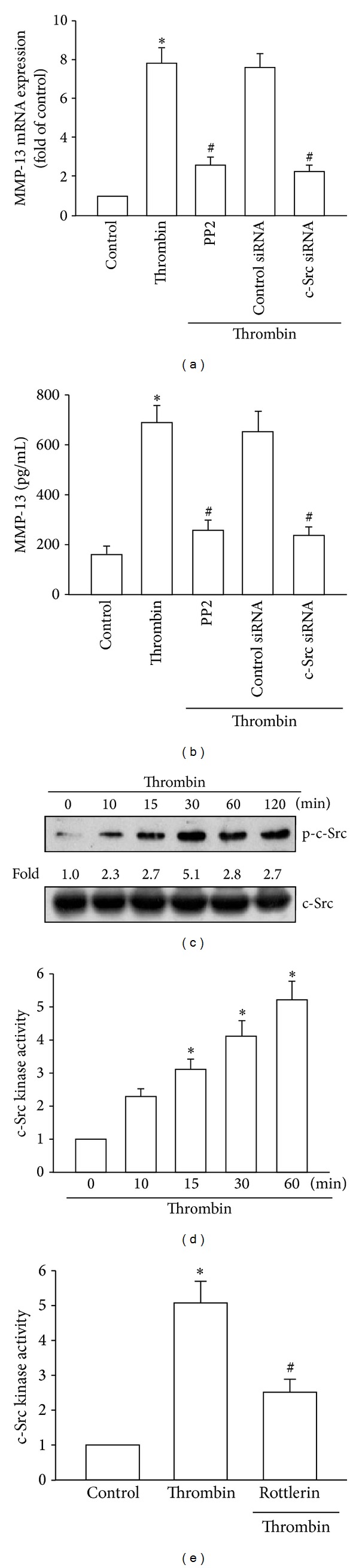
Activation of c-Src in thrombin-mediated MMP-13 expression in chondrocytes. ((a) and (b)) qPCR and ELISA analyses of MMP-13 expression in chondrocytes that were pretreated for 30 min with PP2 (3 *μ*M) or transfected with c-Src siRNA for 24 h and then stimulated with thrombin for 24 h. ((c) and (d)) Western blotting analysis of phosphorylation and kinase activity of c-Src in cells incubated with thrombin for the indicated time intervals. (e) Kinase activity of c-Src in chondrocytes pretreated for 30 min with rottlerin and stimulated with thrombin for 60 min. *P < 0.05 as compared with basal levels. ^#^ *P* < 0.05 as compared with the levels in the thrombin-treated group.

We next examined whether the thrombin-induced increase in PKC*δ* activation subsequently enhanced c-Src activation. Incubation of cells with the c-Src inhibitor PP2 or transfection of cells with c-Src siRNA markedly blocked thrombin-induced MMP-13 expression (Figures 3(a) and 3(b)). Phosphorylation of the Tyr^416^ residue mediates the activation of c-Src [29]. Stimulation of cells with thrombin enhanced c-Src phosphorylation at Tyr^416^ (Figure 3(c)) and promoted c-Src kinase activity (Figure 3(d)). Preincubation of cells with rottlerin reduced the thrombin-stimulated increase in c-Src kinase activity (Figure 3(e)). These results indicate that thrombin increases MMP-13 expression through PAR1/PAR3 and activates PKC*δ*-dependent c-Src activation in human chondrocytes.

### 3.4. Involvement of EGFR Transactivation and PI3K/Akt Pathway in Thrombin Induces MMP-13 Expression in Human Chondrocytes

**Figure 4:**
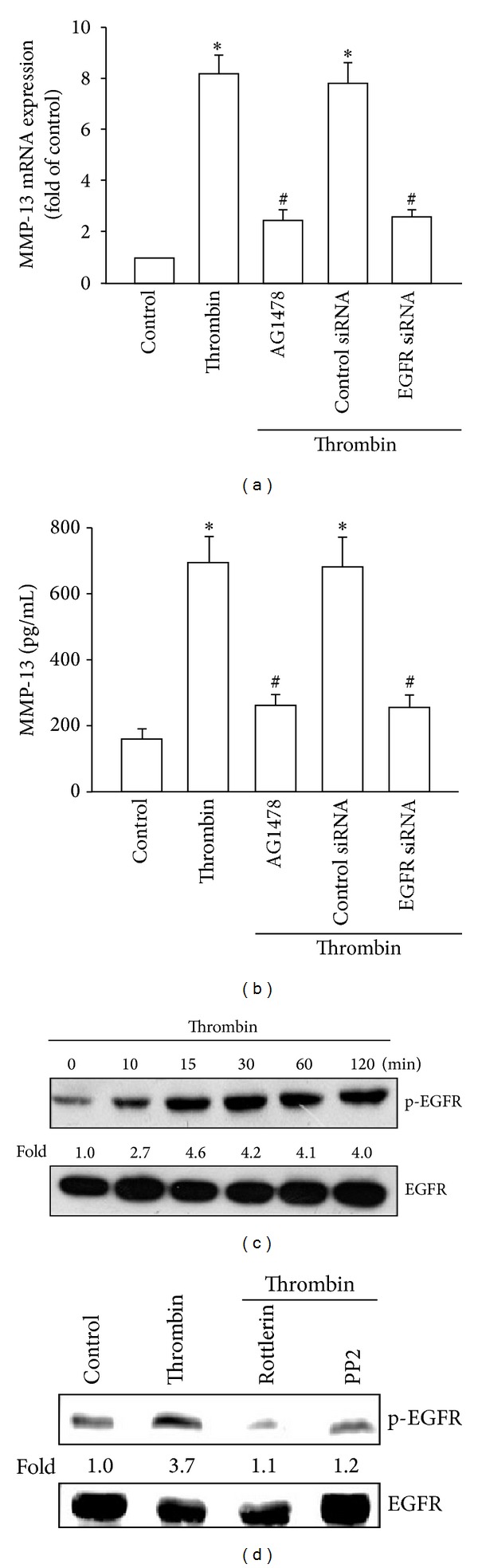
Thrombin-mediated EGFR transactivation in human chondrocytes. ((a) and (b)) qPCR and ELISA analyses of MMP-13 expression in chondrocytes, which were pretreated with AG1478 (1 *μ*M) for 30 min or transfected with EGFR siRNA for 24 h and then stimulated with thrombin for 24 h. (c) Western blotting analyses of EGFR phosphorylation in cells incubated with thrombin for the indicated time intervals. (d) Western blotting analyses of EGFR phosphorylation in chondrocytes pretreated with rottlerin or PP2 for 30 min and then stimulated with thrombin for 60 min. *P < 0.05 as compared with basal levels. ^#^ *P* < 0.05 as compared with the levels in the thrombin-treated group.

EGFR transactivation has been reported to be involved in thrombin-mediated cell functions [25, 30]. Therefore, we used a selective EGFR inhibitor AG1478 to examine whether EGFR transactivation was involved in thrombin-mediated increase in MMP-13 expression. Pretreatment of chondrocytes with AG1478 or transfection with EGFR siRNA diminished the thrombin-mediated increase in MMP-13 expression (Figures 4(a) and 4(b)). The phosphorylation of Tyr^1173^ is essential for EGFR activation [31]. Stimulation of cells with thrombin promoted Tyr^1173^ phosphorylation of EGFR in a time-dependent manner (Figure 4(c)). However, pretreatment of cells with rottlerin or PP2 inhibited the thrombin-mediated increase in EGFR phosphorylation at Tyr^1173^. These results indicated that EGFR transactivation mediated the thrombin-stimulated enhancement of MMP-13 expression in human chondrocytes.

**Figure 5:**
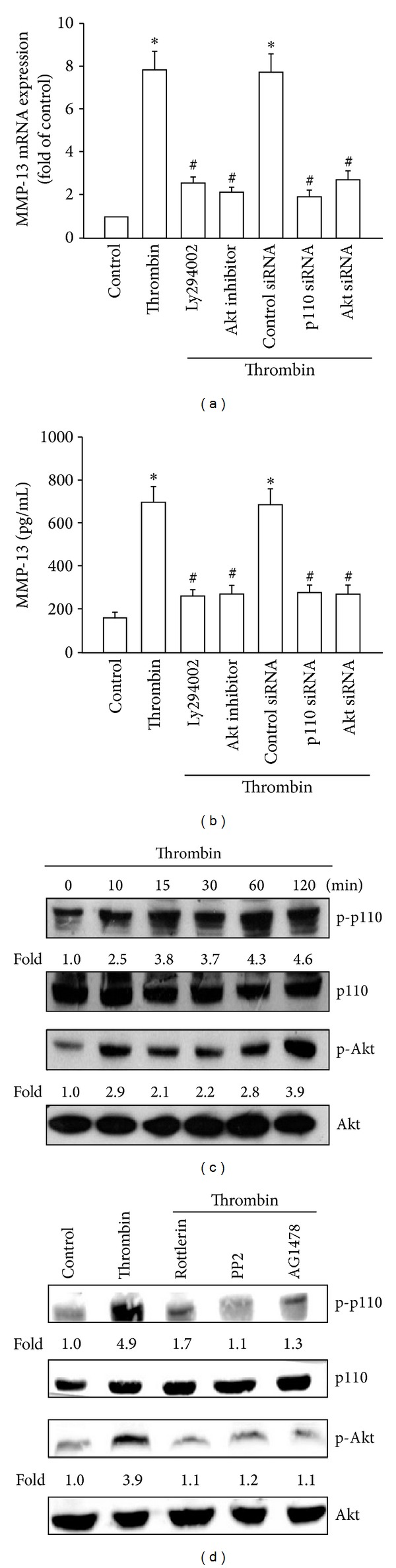
Involvement of EGFR transactivation and subsequent PI3K/Akt activation in thrombin-induced MMP-13 expression. ((a) and (b)) qPCR and ELISA analyses of MMP-13 expression in chondrocytes, which were pretreated with Ly294002 or the Akt inhibitor for 30 min or transfected with siRNAs against p110 or Akt for 24 h and then stimulated with thrombin for 24 h. (c) Western blotting analyses of p110 and Akt phosphorylation in cells incubated with thrombin for the indicated time intervals. (d) Western blotting analyses of p110 and Akt phosphorylation in chondrocytes pretreated with rottlerin, PP2, or AG1478 for 30 min and then stimulated with thrombin for 60 min. *P < 0.05 as compared with basal levels. ^#^ *P* < 0.05 as compared with the levels in the thrombin-treated group.

A previous study has shown that the thrombin-promoted transactivation of EGFR was dependent on the PI3K/Akt signaling pathway [32]. To verify this mechanism in chondrocytes, we analyzed whether thrombin stimulation promoted the EGFR-dependent PI3K/Akt activation. Pretreatment of cells with the PI3K inhibitor Ly294002 or the Akt inhibitor abolished the thrombin-mediated increase in MMP-13 expression (Figures 5(a) and 5(b)). On the other hand, transfection of cells with siRNAs against p110 or Akt led to reduction in the thrombin-stimulated increase in MMP-13 expression (Figures 5(a) and 5(b)). In addition, thrombin stimulation led to phosphorylation of p110 and Akt (Figure 5(c)). Furthermore, pretreatment of cells with rottlerin, PP2, or AG1478 inhibited the thrombin-stimulated p110 and Akt phosphorylation (Figure 5(d)). These results suggest that thrombin induces MMP-13 production through activation of PKC*δ* and c-Src and through EGFR transactivation, which in turn leads to activation of the PI3K/Akt signaling pathway in human chondrocytes.

### 3.5. AP-1 Activation Is Involved in Thrombin-Induced MMP-13 Expression

**Figure 6:**
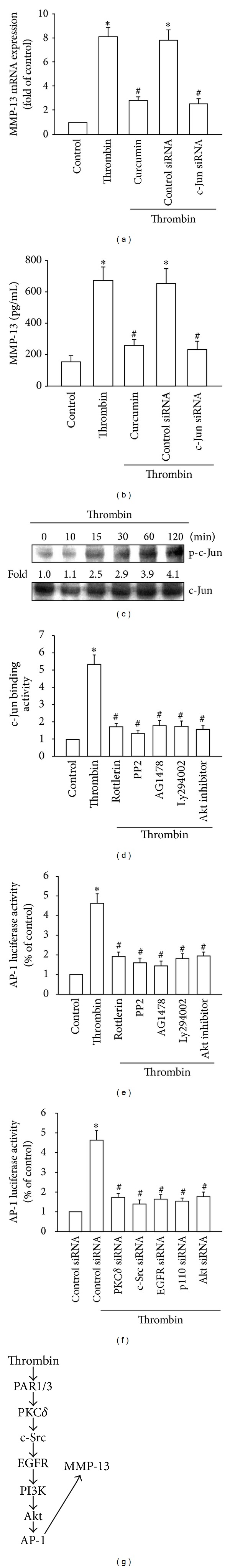
Involvement of AP-1 in the thrombin-induced enhancement of MMP-13 production. ((a) and (b)) qPCR and ELISA analyses of MMP-13 expression in chondrocytes, which were pretreated with curcumin for 30 min or transfected with c-Jun siRNA for 24 h and then stimulated with thrombin for 24 h. ((c) and (d)) Western blotting analyses of c-Jun phosphorylation in cells incubated with thrombin for the indicated time intervals and in cells pretreated with rottlerin, PP2, AG1478, Ly294002, or the Akt inhibitor for 30 min and stimulated with thrombin. ((e) and (f)) Chromatin immunoprecipitation and luciferase activity assays of AP-1 (f). Luciferase activity assays of cells transfected with siRNAs against PAR1, PAR3, PKC*δ*, c-Src, EGFR, p110, or Akt for 24 h and then stimulated with thrombin for 24 h. *P < 0.05 as compared with basal levels. ^#^ *P* < 0.05 as compared with the levels in the thrombin-treated group. (g) Schematic representation of the signaling pathways involved in thrombin-induced MMP-13 expression in human chondrocytes.

The AP-1 binding site has been reported to play an important role in MMP-13 gene expression [33, 34]. Figures 6(a) and 6(b) show that the AP-1 inhibitor curcumin and transfection with c-Jun siRNA abolished thrombin-induced enhancement in MMP-13 production and expression, respectively. On the other hand, stimulation of human chondrocytes with thrombin increased c-Jun phosphorylation in a time-dependent manner (Figure 6(c)). AP-1 activation was further evaluated by analyzing the results of the chromatin immunoprecipitation assays and AP-1 luciferase activity. The *in vivo* recruitment of c-Jun to the MMP-13 promoter was assessed using the chromatin immunoprecipitation assay. *In vivo* binding of c-Jun to the AP-1 element within the MMP-13 promoter occurred after thrombin stimulation (Figure 6(d)). Binding of c-Jun to the AP-1 element by thrombin was attenuated by treatment of chondrocytes with rottlerin, PP2, AG1478, Ly294002, or the Akt inhibitor (Figure 6(d)). To assess AP-1 activation after thrombin treatment directly, cells were transiently transfected with AP-1-luciferase as an indicator of AP-1 activation. As shown in Figure 6(e), thrombin treatment of chondrocytes for 24 h increased AP-1-luciferase activity. In addition, the thrombin-induced increase in AP-1-luciferase activity was reversed in cells treated with rottlerin, PP2, AG1478, Ly294002, or the Akt inhibitor (Figure 6(e)). Cotransfection of cells with siRNAs against PKC*δ*, c-Src, EGFR, p110, or Akt reduced thrombin-enhanced AP-1 luciferase activity (Figure 6(f)). Taken together, these data suggest that activation of PAR1/PAR3 led to activation of PKC*δ*, c-Src, EGFR, PI3K, Akt, c-Jun, and the AP-1 pathway, all of which were required for the thrombin-induced increase in MMP-13 in human chondrocytes.

## 4. Discussion

The synovium of patients with OA and RA contains many types of cytokines and chemokines, such as IL-1, TNF-*α*, and MIP-1, and many types of MMPs [35]. These can induce the breakdown of cartilage. MMP-13 expression has been detected in several pathological conditions that are characterized by the destruction of normal collagen tissue architecture. Therefore, MMP-13 may be a novel target for developing new strategies for treatment of arthritis [8]. Thrombin has been shown to act as a mitogen that stimulates abnormal proliferation and to play an important role in RA and OA pathogenesis [12]. In this study, we identify MMP-13 as a target protein for the thrombin signaling pathway, which regulates breakdown of cartilage. We also show that enhancement of MMP-13 production by thrombin requires activation of PAR1/3 receptor, PKC*δ*, c-Src, EGFR transactivation, PI3K/Akt, c-Jun, and AP-1 signaling pathways.

Thrombin is known to activate 3 PARs PAR1, PAR3 and PAR4 [36]. However, our data suggests that PAR1 and PAR3, but not PAR4 receptors were required for thrombin-induced MMP-13 expression. Incubation of cells with GYPGOV-NH_2_ (PAR4 agonist peptide) did not enhance the MMP-13 production in chondrocytes. In addition, thrombin-induced MMP-13 expression could not be reduced by transfection of cells with PAR4 siRNA. Therefore, PAR1 and PAR3 receptors mediated the thrombin-stimulated increase in MMP-13 expression in chondrocytes. In addition to gene expression, a similar receptor signaling mechanism has also been reported in the thrombin-induced HO-1 expression in human synovial fibroblasts, which were through PAR1/PAR3 receptors [30], and in thrombin regulated expression of MMPs and cell migration in chondrosarcomas [37]. In contrast, it has been reported that thrombin increased IL-6 production in human synovial fibroblasts through the PAR1 receptor [38]. We previously reported that PAR1 but not PAR3 was involved in the thrombin-mediated CCL2 expression in osteoblasts [17]. Therefore, in various cell types thrombin acts via different PAR receptors to induce different gene expressions to regulate cell functions.

PKC*δ*-dependent c-Src activation has been shown to regulate thrombin-mediated cell functions [25, 27]. In the current study, we found that the specific PKC*δ* inhibitor rottlerin and the c-Src inhibitor PP2 abolished the thrombin-mediated potentiation of MMP-13 expression, suggesting that activation of PKC*δ* and the activation of c-Src were obligatory events in thrombin-induced MMP-13 expression in human chondrocytes. This was confirmed by siRNA experiments in which the thrombin-induced MMP-13 expression was diminished by transfection of cells with PKC*δ* and c-Src siRNA. In addition, treatment of chondrocytes with thrombin also induced PKC*δ* or c-Src phosphorylation and kinase activity. These effects were inhibited by rottlerin, indicating involvement of PKC*δ*-dependent c-Src activation in thrombin-mediated MMP-13 production.

It has been reported that transactivation of EGFR and subsequent enhancement of PI3K/Akt activation mediated the signaling in response to thrombin [32, 39]. In this study, treatment of cells with EGFR, PI3K and Akt inhibitor or transfection of cells with EGFR, PI3K, and Akt siRNAs reduced the thrombin-mediated MMP-13 expression. Furthermore, we also found that thrombin increased EGFR, p110, and Akt phosphorylation. However, PKC*δ*, c-Src, and EGFR inhibitors inhibited thrombin-mediated p110 and Akt phosphorylation. These results suggest that thrombin induced MMP-13 production through PKC*δ*, c-Src, and EGFR transactivation and via the PI3K/Akt signaling pathway in human chondrocytes.

Previous reports indicate that AP-1 controls induced transcription of MMP-13 in human chondrocytes [9]. The results of our current study show that AP-1 activation contributes to thrombin-induced MMP-13 expression in human chondrocytes. Pretreatment of cells with an AP-1 inhibitor curcumin reduced the thrombin-induced MMP-13 expression. Therefore, the AP-1 binding site is likely to be the most important site for thrombin-induced MMP-13 production. Members of the Jun and Fos families of transcription factors bind to the AP-1 sequence. These nuclear proteins interact with the AP-1 site as Jun homodimers or Jun-Fos heterodimers formed by protein dimerization through their leucine zipper motifs [40]. The results of our study show that thrombin induces c-Jun phosphorylation, whereas c-Jun siRNA abolished thrombin-induced MMP-13 expression in human chondrocytes, indicating that c-Jun activation mediates the thrombin-induced increase in MMP-13 expression. Furthermore, thrombin increased the binding of c-Jun to the AP-1 element within the MMP-13 promoter, as shown by the results of the chromatin immunoprecipitation assay. Binding of c-Jun to the AP-1 element was attenuated by rottlerin, PP2, AG1478, Ly294002, and Akt inhibitor. By using transient transfection of AP-1-luciferase as an indicator of AP-1 activity, we also observed that thrombin induced an increase in AP-1 activity, which was reduced by rottlerin, PP2, AG1478, Ly294002, or Akt inhibitor. These results indicate that thrombin probably acts through PAR1/3, PKC*δ*, c-Src, and EGFR transactivation and the PI3K, Akt, c-Jun, and AP-1 pathways to induce MMP-13 production in human chondrocytes.

In this study, we determined the signaling pathways involved in thrombin-induced MMP-13 expression in human chondrocytes. We found that thrombin augmented MMP-13 expression by binding to the PAR1/PAR3 receptor and activating PKC*δ* and c-Src and through EGFR transactivation, which in turn leads to PI3K and Akt activation, thereby enhancing binding of c-Jun to the AP-1 site and resulting in increased MMP-13 expression (Figure 6(g)). The discovery of this thrombin signaling pathway helps us understand the mechanism underlying arthritis pathogenesis, which may lead to the development of effective therapies in the future.
